# Basal body docking in airway ciliated cells

**DOI:** 10.18632/oncotarget.4609

**Published:** 2015-06-23

**Authors:** Feng-Qian Li, Saul S. Siller, Ken-Ichi Takemaru

**Affiliations:** Department of Pharmacological Sciences, Stony Brook University, Stony Brook, NY, USA

**Keywords:** Immunology and Microbiology Section, Immune response, Immunity

Multiciliated cells (MCCs) in the respiratory epithelium play crucial roles in the innate defense system against airborne pathogens and pollutants. Each MCC possesses 200-300 cilia on their apical surface, which beat in a synchronized manner to clear mucus with foreign particles from the respiratory tract. Ciliary dysfunction leads to serious respiratory diseases including chronic infection as seen in patients with primary ciliary dyskinesia (PCD), a ciliopathy with defective multicilia. However, the molecular mechanisms underlying the differentiation of MCCs are poorly understood.

In MCC precursor cells, downregulation of Notch signaling induces expression of the coiled-coil protein Multicilin (Mcidas), which in turn upregulates expression of the ciliogenesis transcription factors Myb, RFX and FoxJ1 that direct MCC differentiation. The majority of centrioles are generated *de novo* via an acentriolar pathway that involves centriole nucleation from fibrogranular structures called deuterosomes. In contrast, a canonical centriolar pathway, in which centrioles replicate using the existing centriole as a template, contributes to a small portion of the total centriole population. Nascent centrioles mature by acquiring accessary structures, which include distal appendages (DAs). Upon docking to the apical membrane, centrioles and DAs transform into basal bodies and transition fibers (TFs), respectively. DAs/TFs are fibrous structures that emanate from the distal end of the B-tubules of each of the nine centriole/basal body triplet microtubules, and are important for membrane docking [1]. At the early stages of ciliogenesis, small vesicles termed DA vesicles (DAVs) are captured by DAs and then fuse with each other to form a large membranous cap, the so-called ciliary vesicle, which later fuses with the apical membrane to facilitate basal body docking [1, 2], although alternative pathways may also exist. The extension of cilia from the basal bodies is mediated by the intraflagellar transport (IFT) system.

Chibby (Cby) is an evolutionarily conserved 15-kD coiled-coil protein that localizes to the base of cilia. We previously reported that Cby−/− mice suffer from chronic respiratory infection due to a lack of effective mucociliary transport [3]. Consistent with this phenotype, we further demonstrated that Cby−/− MCCs show a marked reduction in the number of cilia and consistently contain undocked basal bodies in the apical cytoplasm. Taken together, these data suggested that Cby is required for efficient basal body docking. However, the precise molecular function of Cby during MCC differentiation remained unknown.

Recently, using SIM and STORM super-resolution microscopes, we found that Cby protein clusters at the base of mature cilia as a ring with a diameter of 300 nm and a height of 100 nm [4]. Intriguingly, immuno-EM revealed that Cby is enriched around the distal portion of TFs where the TFs contact the apical membrane. During early stages of MCC differentiation, Cby localizes to the DAs of migrating centrioles, which are the sites of DAVs recruitment. Notably, in Cby−/− MCCs, only small DAVs are bound to the DAs, which indicates that Cby is not essential for the DAV-DA attachment. Rather, Cby appears to be required for the subsequent fusion of DAVs into larger ciliary vesicles. Molecularly, we demonstrated that Cby is recruited to the DAs through a physical interaction with the DA protein CEP164. Cby then associates with the membrane trafficking component Rabin8, a guanine nucleotide exchange factor (GEF) for the small GTPase Rab8, to promote recruitment of Rab8 and efficient assembly of ciliary vesicles.

Since the first morphological EM study on MCCs in rat lungs in the 1960s [2], the molecular basis for basal body docking has remained largely elusive. Our study revealed that Cby facilitates basal body docking by promoting proper formation of ciliary vesicles. Our findings also underscore the biological importance and requirement of ciliary vesicle formation for efficient basal body docking. We postulate that the ciliary vesicle system is particularly important for MCC differentiation and ciliogenesis as they must coordinate the docking of hundreds of basal bodies over a short period of time. Cby most likely mediates ciliary vesicle assembly during primary ciliogenesis in a similar manner as Cby depletion significantly reduces the number of primary cilia in various cell types [5, 6].

We are only beginning to understand the molecular details for ciliary vesicle assembly. Recently, the membrane shaping proteins EHD1 and EHD3 and the SNARE membrane fusion regulator SNAP29 have been shown to function in ciliary vesicle formation [7]. However, the relationship between Cby and these novel regulators is currently unclear. Further identification and characterization of DA components and their associated proteins are critical for broadening our understanding of ciliogenesis and the pathobiology of ciliopathies.
